# Design and development of PI controller for DFIG grid integration using neural tuning method ensembled with dense plexus terminals

**DOI:** 10.1038/s41598-024-56904-7

**Published:** 2024-04-04

**Authors:** R. R. Hete, Tarun Shrivastava, Ritesh Dash, L. Anupallavi, Misba Fathima, K. Jyotheeswara Reddy, C. Dhanamjayalu, Faruq Mohammad, Baseem Khan

**Affiliations:** 1 Department of Electrical Engineering, G.H.Raisoni University, Amravati, India; 2 Department of Electrical Engineering, G. H. Raisoni Institute of Engineering and Technology, Nagpur, India; 3 School of Electrical and Electronics Engineering, REVA University, Bangalore, India; 4 School of Electrical Engineering, Vellore Institute of Technology, Vellore, India; 5 Department of Chemistry, College of Science, King Saud University, P.O. Box 2455, 11451 Riyadh, Kingdom of Saudi Arabia; 6 Department of Electrical and Computer Engineering, Hawassa University, Hawassa 05, Ethiopia

**Keywords:** Energy science and technology, Engineering

## Abstract

In a DFIG grid interconnected system, the control of real and reactive power relies on various factors. This paper presents an approach to regulate the flow of real and reactive power using a Neural Tuning Machine (NTM) based on a recurrent neural network. The focus is on controlling the flow of reactive power from the rotor-side converter, which is proportional to the grid-side controller through a coupling voltage. The proposed NTM method leverages neural networks to fine-tune the parameters of the PI controller, optimizing performance for DFIG grid integration. By integrating dense plexus terminals, also known as dense connections, within the neural network, the control system achieves enhanced adaptability, robustness, and nonlinear dynamics, addressing the challenges of the grid. Grid control actions are based on the voltage profile at different bus locations, thereby regulating voltage. This article meticulously examines the analysis in terms of DFIG configuration and highlights the advantages of the neural tuning machine in controlling inner current loop parameters compared to conventional PI controllers. To demonstrate the robustness of the control algorithm, a MATLAB Simulink model is designed, and validation is conducted with three different benchmarking models. All calculations and results presented in the article strictly adhere to IEEE and IEC standards. This research contributes to advancing control methodologies for DFIG grid integration and lays the groundwork for further exploration of neural tuning methods in power system control.

## Introduction

The increase in population and global energy demand has drawn the attention of many energy practitioners to produce clean and green energy to meet the peak demand. Sustainable development of clean energy resources not only creates a friendly environment for every individual but also helps to achieve global energy demand. In this regard distributed energy resources such as Solar PV, Wind mill and Geothermal energy play a very vital role. However, extracting power from wind energy systems is gaining more popularity among all other types of resources. As of today, the total wind energy capacity of India is 43.7 GW. This shows that there is a huge potential for wind energy, however, the popularity in standard is limited because of the complexity of holding control and management^1^.

The DFIG control action generally consists of two interdependent control actions such as the Rotor side controller (RSC) and Grid side controller (GSC). The stability operation requires a thorough balance between RSC and GSC^2^. Different control actions consisting of two independent loops, that is Inner current control loop and Outer voltage control loop based on conventional PI control, Fuzzy PI controller and ANFIS PI controller have been studied in the literature. Some of the modern PI control techniques to control the reactive power based on direct power control, fractional order control and sector field scale control have been proposed in the literature. The power quality issues consisting of the classical PI technique suffer from large torque with current ripple and variable switching frequency^3,4^. All these things will have a negative impact on the switches of converter which generally incurs excess switching losses in GSC and RSC. The change in rotor flux current leads to the development of Space vector modulation (SVM) has been developed. One of the disadvantages of Space vector modulation is a small change in DC link voltage which may affect the performance of the voltage matrix in SVM. SVM also increases the switching loss. The change in DC link voltage because of changes in voltage across the coupling capacitor is a common parameter related to variations in wind velocity^5^.

Fractional order (FO) controller-based rotor side converter technology has played a vital role in tuning the $K_P$ and $K_I$ values for an FO-PI controller^6^. This is because the FO-PI controller has five tuning parameters against two tuning parameters in a conventional PI controller. The FO controller uses the concept of personal derivatives with fewer parameters to optimize and evaluate the reference values for the inner current control loop. Similarly, the outer voltage control loop can be tuned based on the requirement of reactive power, that is to be injected into the grid. However, the disadvantage associated with the FO-PI controller is the generation of more harmonics content at the output which increases the voltage at the Point of common coupling (PCC)^7^.

Starter frequency control in a DFIG requires control of the speed of wind turbines which in turn depends on the rotor pitch angle. Controlling the rotor pitch angle is not only the parameter which effects the performance of DFIG, some other parameters such as blade angle, hub, gear teeth ratio, frequency of the grid to which the DFIG is connected and the output voltage. To integrate all these parameters in the PI tuning system Fuzzy Logic Controller (FLC) has been investigated. The advantage of FLC is that it does not require the mechanical parameters of the DFIG to decide the tuning parameters of the PI controller. The FLC can handle different power quality disturbances such as variation in frequency, voltage ride-through capability, fault ride-through capability and thereby making the system more user-friendly by integrating into the conventional grid system^8,9^.

The Authors in^10^, have proposed a Hidden Layer Recurrent Neural Network (HLRNN) for designing and developing the controller based on PI-Control scheme. They have applied the Indirect Vector Control scheme to implement the model and its associated logic. The proposed model not only enhances the self-adaptability of the PI controller but has also shown a track record of improved response time, minimization of static error and overshoot in the system performance. Although the model has several advantages but becomes sluggish response to variation in wind speed conditions. In^11^ the authors proposed an integral backstepping nonlinear controller for controlling three parameters such as grid level exchange power and bus capacitor voltage. The technique utilizes the Lyapunov function stability for backstepping control law. The model has been tested using a matlab Simulink model. Maximum power point strategies in an explicit manner have been presented. However, the stability concern over other models particularly under grid disturbance model has not been presented.

Mathematical modeling of DFIG wind turbine using PR controller has been proposed by author^12^. The proposed PR controller has been tested under grid harmonics and its correlation to generator torque. The PR controller is the major factor in eliminating the negative sequence from the harmonic component of rotor part. The proposed model has been investigated through experimental setup. In the paper^13^ the authors have proposed about the coupling of DFIG-WT with the grid and have investigated the performance under voltage imbalance conditions. The author is more concerned about the environmental impact and corresponding utilization of the wind energy system. The proposed Naslin polynomial technique for PI controller gains in removing the negative sequence component from the rotor current has been demonstrated. Although the proposed controller is robust in removing the negative sequence component from the rotor but its application becomes invariant to changes in different wind velocity and wind speed. Again, discussion about the performance of DFIG during grid imbalance conditions has not been investigated.

The regulation of active and reactive power with unit power factor using Back stepping control for wind power plant with adaptive weighted PSO has been investigated by authors^14^. The model presents a comparative analysis between Back stepping and PI control strategies. The robustness and fast response analysis has been carried out by using matlab Simulink model. The adaptive weighted PSO has shown best performance under certain conditions. However, the complexity increases with increase in the generator parameters and varying wind speed configurations.

Adaptive Neural Fuzzy Inference System (ANFIS) is superior as compared to the fuzzy-enabled system, it is because the ANFIS uses IF-THEN rules to determine the adaptability of the non-linear cost function involved in approximating the gain parameters. The membership function which determines the controllability of the tuning parameters is dynamic instead of static as in FLC^15^.

Vector control ANFIS model tp tune the $K_P$ and $K_I$ of RSC, which have has been investigated in the literature. The coupling voltage between GSC and RSC has been taken into consideration and referenced for deciding the vector. It is understood that during the fault condition, there will be a small to large fluctuation, for both voltages will occur at the coupling capacitor. These in turn change the decision of $K_P$ and $K_I$, even if there is an incipient fault^16–18^.

Adaptive Neural Network (ANN) based PI controller has been investigated by many researchers. The back-propagation algorithm has been used to decide the weight function of each neural network. The efficiency of the ANN-based PI controller depends on the training of each neuron and the number of hidden layers present in the system^19^. The complexity of the ANN-PI controller can be reduced with a reduction in the number of hidden layers and cross co-relation. In most of the literature study, it is observed that authors are limited to a maximum of five hidden layers. The transient analysis of ANN-enabled PI controller has reported better performance during grid synchronization and under faulty conditions. The disadvantage associated with ANN fuzzy or ANFIS model is that there is no impact of past data on tuning the performance of gain parameters under dynamic sliding mode. To avoid all such conditions, researchers are focusing more on the real-time estimation of parameters with changing loading conditions at the output of DFIG grid interconnected system^20^. In this regard, machine learning approaches are taking initiation in configuring the time series-based pattern of different loading configurations and the behaviour of gain sequence. These two parameters when combined, the evaluation of different gain can be made dynamic which will act in the PI controller as and when required^21^.

The data for the machine learning approach can be configured either based on seasonal variation or by making the configuration static. LSTM-enabled models have been investigated by many researchers in the past to showcase the effect of seasonal patterns in configuring the gain for the PI controller^22^. One of the disadvantages associated with LSTM is the requirement of a large memory set and the activation function used for the optimization. This problem can be addressed by using a recurrent neural network-based Neural Tuning Machine (NTM). This method provides the advantage of both fuzzy pattern and ANN capabilities to solve the complex problem. The use of external memory makes it easier to reduce the computational burden on system configuration. The data patterns used in NTM are differentiable thereby optimizing the search engine with gradient descent^23^. Based on the pre-feasibility study it is understood that the NTM along with PI controller can provide higher efficiency as compared to other models such as classical PI, ANFIS-PI and ANN-PI. The objectives of the research work can be summarised as follows,

- Design and development of closed-loop inner current control loop using NTM-PI controller for Rotor Side Controller (RSC)
- Design an outer voltage control loop using ANN-based NTM and optimise the tuning parameters with respect to different loading configurations. Three types of loading configuration have been taken into consideration, such as $100\%$ loading, $75\%$ loading and $50\%$ loading pattern.
- Validating the proposed NTM model with the standard bench-marking model strictly adhering to IEEE standards.

In lieu of the above objectives, the proposed research work focuses on novel application of neural network ensembles for PI controller design in DFIG grid integration. It includes a collective convergence of different multiple neural networks based PI controller in designing the DFIG grid interconnection system, which potentially increases the performance of individual network and thereby increasing the overall system performance. Again, the interconnection of dense plexus terminals increases the learning capabilities of the network, thereby providing the superior PI controller tuning characteristics over conventional PI controller. In the ongoing discussion, a detailed literature survey concerning the DFIG grid interconnection system with various controlling architectures has been conducted. Three notable objectives derived for the present research have been outlined above. The section ’DFIG Modelling’ includes the mathematical formulation of the identified problem related to power flow for both DFIG and inverter-based control actions. In the ’Neural Tuning Machine’ section, the NTM optimization procedure is discussed. The ’Bench-Marking Models Performance Evaluation’ section analyzes three benchmarking models used to evaluate the performance of the proposed model. The ’Result Analysis’ section presents the MATLAB-based modeling of the proposed NTM-controlled architecture, followed by the results and discussion.

## DFIG modelling

The wind turbine’s output power is based on the function of three inputs such as wind velocity, power generator, wind gust and transmission and distribution. Voltage sag, voltage swell and harmonics are the other terms through which power quality can be evaluated. In distribution networks generally, the power quality disturbances are introduced by the wind generator. By the use of a direct connection of the induction generator to the grid system, the operation wind generation system is carried out. This is one of the simplest methods. The various advantages of using an induction generator are cost-effectiveness and robustness. In order to inject the real power into the grid, reactive power for the magnetization of the induction motor is required. At the converter terminal, to control the production of active power, a regulated voltage control system is required^24,25^.

In the induction generator, the slip and speed fluctuations are kept at minimum during normal steady-state conditions. In this scenario, the machine’s reactive power is minimal but to increase in the power and load will also increase the motor’s reactive power consumption and motor slip. The equations for stator voltage and flux can be written as

1 $$\begin{aligned} {\left\{ \begin{array}{ll} V_{s} = R_{s}L_{s} + \frac{d\phi _{s}}{dt} \\ \phi _{s} = L_{s}I_{s} + L_{m}I_{r} \end{array}\right. } \end{aligned}$$

Also, the equations for rotor side voltage and flux are given as:

2 $$\begin{aligned} {\left\{ \begin{array}{ll} V_{r}= R_{r}I_{r}+\frac{d\phi _{r}}{dt} - j\omega _{m}\phi _{r}\\ \phi _{r}= L_{r}I_{r}+L_{m}I_{s} \end{array}\right. } \end{aligned}$$

From the above two equations, the rotor and stator voltage can be written as:

3 $$\begin{aligned} {\left\{ \begin{array}{ll} V_{r}= R_{r}I_{r}+ Sj\omega _{s}L_{\sigma r} + Sj\omega _{s}L_{m} (I_{r}+I_{s}) \\ V_{s}= R_{s}I_{s}+ j\omega _{s}L_{\sigma r}I_{s} + j\omega _{s}L_{m} (I_{r}+I_{s}) \end{array}\right. } \end{aligned}$$

In the above equation, “S” is the slip of the system. And from equation 3, active power of rotor and stator becomes,

4 $$\begin{aligned} {\left\{ \begin{array}{ll} P_{s}= \frac{3}{2}R_{e}(V_{s}I_{s})=\frac{3}{2}R_{s} I_{s}^2+\frac{3}{2}\omega _{s}L_{m}R_{s}(j(I_{r}I_{s})) \\ Q_{r}= \frac{3}{2}R_{e}(V_{r}I_{r})=\frac{3}{2}R_{r} I_{r}^2+\frac{3}{2}S\omega _{s}L_{m}R_{s}(j(I_{s}I_{r})) \end{array}\right. } \end{aligned}$$

Here, three phase line voltage can be written as:

5 $$\begin{aligned} {\left\{ \begin{array}{ll} V_{ag} = I_{a}R_{a} + L \frac{dI_{a}}{dt} + V_{fa} \\ V_{bg} = I_{b}R_{b} + L \frac{dI_{b}}{dt} + V_{fb} \\ V_{cg} = I_{c}R_{c} + L \frac{dI_{c}}{dt} + V_{fc} \end{array}\right. } \end{aligned}$$

From Eq. 5,dq component can be derived

6 $$\begin{aligned} {\left\{ \begin{array}{ll} V_{d}=I_{d}R+L\frac{dI_{d}}{dt}-L\omega I_{q}+V_{fd}\\ V_{q}=I_{q}R+L\frac{dI_{q}}{dt}+L\omega I_{d}+V_{fd} \end{array}\right. } \end{aligned}$$

From Eq. 6, three different challenging features can be pointed out such as decoupling item, correction factor and voltage compensation. Therefore, reference current control loop equation becomes

7 $$\begin{aligned} {\left\{ \begin{array}{ll} I_{d}^*= \frac{P^*V_{fd} + Q^*V_{fd}}{V_{fd}^2 + V_{fq}^2} \\ I_{q}^*= \frac{P^*V_{fq} - Q^*V_{fd}}{V_{fd}^2 + V_{fq}^2} \end{array}\right. } \end{aligned}$$

To obtain unity power factor, $I_q$ $^*=0$ and hence,

8 $$\begin{aligned} \begin{aligned} \theta =tanh\frac{V_{\beta }}{V_{\alpha }} \end{aligned} \end{aligned}$$

Power converter voltage is proportional to DC reference voltage and therefore,

9 $$\begin{aligned} {\left\{ \begin{array}{ll} V_{d}=mV_{dc}cos\theta \\ V_{q}=mV_{dc}sin\theta \end{array}\right. } \end{aligned}$$

Therefore, from Eqs. 6, 8 and 9, the power balance equation becomes:

10 $$\begin{aligned} {\left\{ \begin{array}{ll} P = \frac{3}{2} (V_{d} I_{d} + V_{q} I_{q}) \\ i_{dc} = \frac{3}{2} m(i_{d} cos\theta - i_q sin\theta ) \end{array}\right. } \end{aligned}$$

## Neural tuning machine

Neural Tuning machine (NTM) is a type of recurrent neural network-based optimization algorithm, however, it holds the advantage of neural network for pattern recognition and computational models with enough time and memory to solve complex problems. The adaptive behaviour of the NTM makes it different from the recurrent neural network in terms of real-time assessment of controller behaviour. Basically, the NTM consists of two main parts such as a neural network controller and an external memory bank. The memory bank serves as a reference to the previous state of operation and acts as a back-supporting agent for decision disbursement. The content-based searching mechanism makes it more suitable for implementing it along with the PI controller in DFIG grid interconnected system, however, such space varies unconditionally because the output of DFIG greatly depends upon the input factors such as wind velocity, temperature and wind pressure. Therefore a complex algorithm can be designed for NTM by incorporating the transient behavior of the loading pattern at the grid side controller and the intermittent nature of the wind system through the rotor side converter.

To start the NTM procedure it is required to process all the data by aligning them within [1, 0]. In order to achieve this Min–Max normalization method has been applied considering $X_{min}$ as the minimum value in the data set and $X_{max}$ as the maximum value of the data set. The formula used for such an operation is presented below,

11 $$\begin{aligned} X_{n}=\frac{X-X_{min}}{X_{max}-X_{min}} \end{aligned}$$

Handling the window size in NTM-based optimisation requires shorter-length data. Therefore the time series data for $K_P$ and $K_I$ has been cut into smaller window sizes by using the sliding window technique. This procedure acts as a pre-requisition to NTM modeling.

At each time interval T, the PI controller contacts the RSC and GSC to share their captured parameters from DFIG and grid side respectively. This initiation brings autonomy to the system by updating their individual memory in line with the system parameters. To avoid the instability condition during the data processing phase, the system uses LSTM label of work without affecting the present $K_P$ and $K_I$ value. Thus the system continuously learns from the parameters extracted and re-written into the memory bank of the individual controller. In this way, the Grid Side Controller (GSC) regulates the DC linkage voltage thereby controlling the reactive power at the output terminal.

The discussed Dense Plexus Terminal is a fully connected layer usually being characterized by an inter connection pattern between the neuron setup. This allows the layer for an enhanced information exchanged potentials leading to improved learning capability of the system and a robust controller. Several ensembles techniques like bagging, boosting and stacking are also there, however, the Dense Plexus Terminal provides a linear complex meta- learner pattern for rich information exchange.

Based on the above discussion the following items have been proposed to achieve in the model.

- Based on the operating conditions, the data-driven methodology enables the controller to retrieve data from past files and adjust the controller parameters accordingly to achieve zero error during execution. This leads to improvements in three parameters of the control system: reduced settling time, decreased overshoot, and minimized steady-state error over the time series data.
- The proposed neural network can adapt and adjust the gain value of the PI controller in response to real-time demands of various power quality issues. This reduces the need for human effort in tuning the controller’s gain, ultimately enhancing its effectiveness.
- The non-linear characteristics of the wind and DFIG can be taken into consideration for designing the hyper parameter tuning of the controller under different wind velocity by considering the complexity of the system.

## Bench-marking models performance evaluation

DFIG grid-connected system will regulate the output voltage and frequency depending upon the available wind energy at the input side of the turbine. In order to achieve good quality power at the terminal of DFIG it is required to tune the PI controller gain parameters in such a manner that the output must follow the standard IEEE values without compromising the DISCOM’s guidelines for renewable grid interconnection code for wind energy. In this connection, the three bench-marking models have been evaluated separately with three different loading conditions as presented in section I.

### PI controller analysis

The classical PI controller plays a vital role in designing the gain for the inner and outer control loops. The Ziegler–Nichols has been adopted to determine the optimal value for $K_P$ and $K_I$. The transient analysis with step input has been applied to the derived transfer function for checking the robustness of the control. The detailed gain parameters for the inner current control loop have been presented in Table 1.

**Table 1:** PI controller gain for inner current control loop and transfer function performance.

S.N.	Parameter	Magnitude	Remarks
01	Proportional gain	0.83 [0.81 0.82]	NA
02	Integral gain	5	NA
03	Rise time	0.17	ms
04	Peak time	0.174	ms
05	Delay time	0.021	ms (sluggish response)
06	Setting time	0.23	ms
07	Maximum overshoot	17.08	%
08	Repose time	6.53	ms (sluggish response)

As noticed from Table 1 the proportional gain is 0.85 and that of integral gain is 5. This shows that the upper boundary for proportional gain will be 0.87 and that of the lower boundary is 0.81. Similarly, the integral gain is 5. The transfer function performance (Derived from curve fitting) for rise time and peak time is 0.17 and 0.174 ms. These times show that the system has relatively low-performance subject to sudden changes in loading conditions at the output of the DFIG. The delay and settling time analysis is respectively 0.021 and 0.023 ms. A maximum overshoot of $17.08\%$ has been noticed which leads to an under-damped system with four oscillations before settling at 0.23 ms. The overshoot represents the inner current control loop will suffer from any kind of transient disturbance by a $\%$ of 17.08. Similarly, the response time for the inner current loop using the PI control technique is 6.53 ms (Sluggish response).

**Table 2:** PI controller gain for outer voltage control loop and transfer function performance.

S.N.	Parameter	Magnitude	Remarks
01	$K_P$	0.87	NA
02	$K_I$	5.2	NA
03	Rise time	0.27	ms
04	Peak time	0.281	ms
05	Delay time	0.084	ms
06	Setting time	0.33	ms
07	Maximum overshoot	19.27	%
08	Response time	5.80	ms (sluggish response)

Table 2 represents PI controller gain for the outer voltage control loop and transfer function performance. The outer voltage control loop is responsible for reactive power compensation at the converter loop. As observed the response time is 5.80 ms as compared to a response time of 6.53 ms in Table 1. This represents the outer voltage control loop using the PI controller is a little bit faster in performance

**Figure 1:**
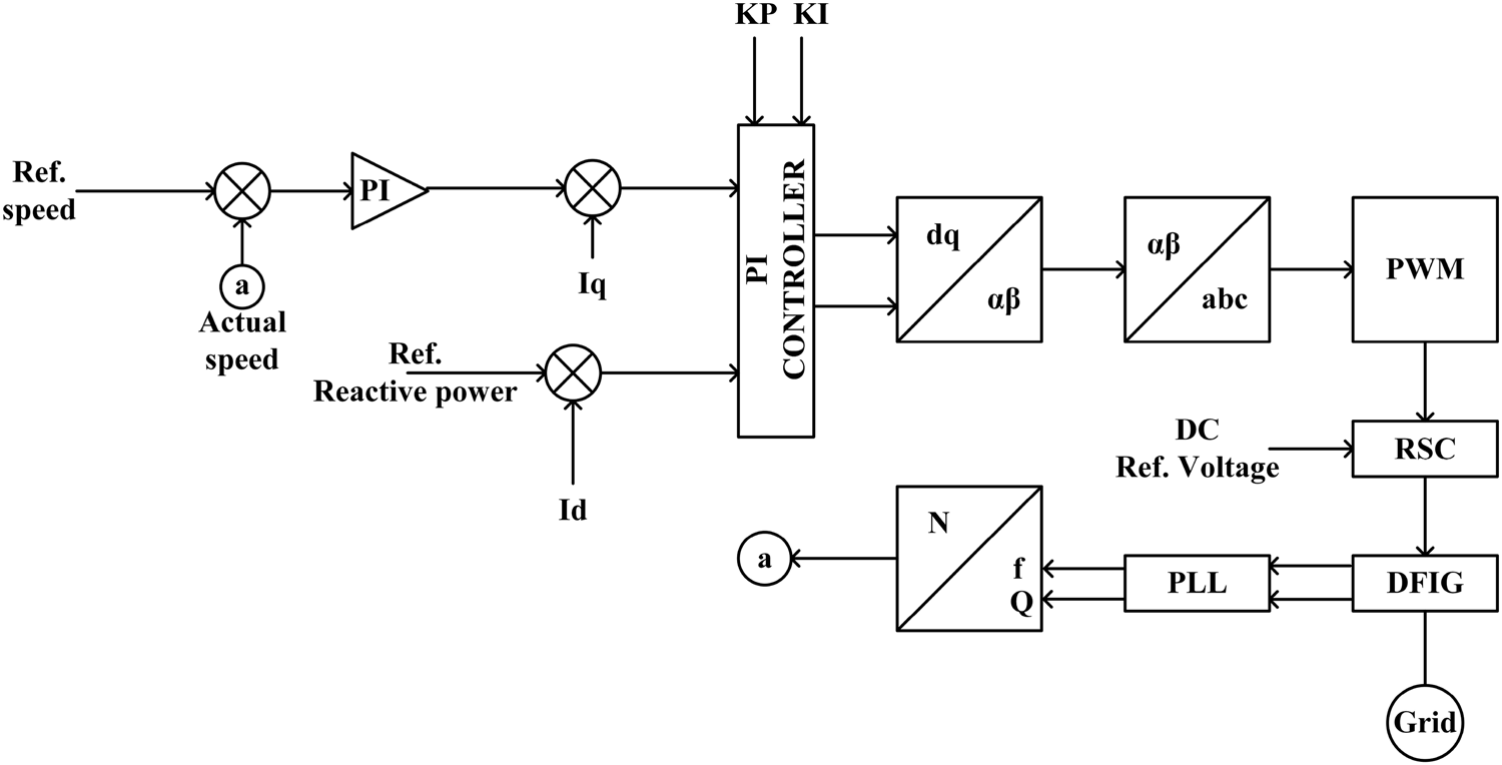
Block diagram of PI-controlled DFIG grid interconnected system.

Figure 1 represents the block diagram of PI controlled DFIG grid interconnected system. As noticed the reference speed and actual speed have been taken at the output of DFIG by using a Phase Locked Loop (PLL) for frequency and phase angle respectively. The rotor side controller will act on DC reference voltage by using the PWM converter as a derived parameter of the PI controller. The $I_d$ and $I_q$ will be derived using Park’s Transformation to control the respective reactive and active power at the terminal of the DFIG inverter system. Both $K_P$ and $K_I$ were tuned using the Ziegler–Nichols method. The detailed result analysis is presented under section v.

### ANFIS controller

**Figure 2:**
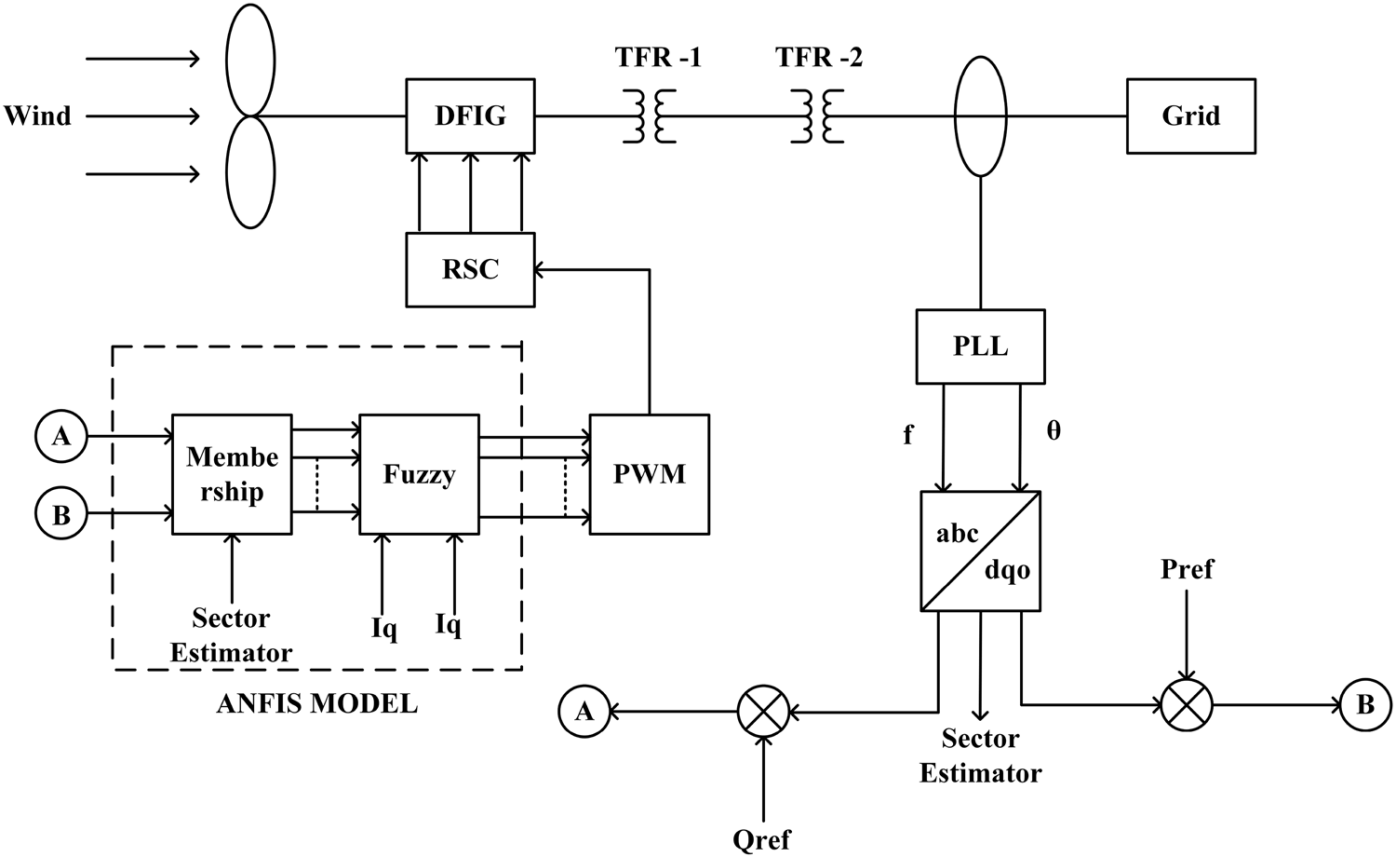
Block diagram of ANFIS controlled DFIG grid interconnected system.

The modified version of the PI controller using ANFIS is presented in this section. Two parameters which have been taken into consideration for ANFIS are $I_d$ and $I_q$ values, generated from Park’s Transformation. The ANFIS model consists of three hidden layers with an IF-THEN statement using a triangular membership function. Here five types of membership functions such as NL, NS, ZE, PS and PL (Refer. Fig. 4b) are used. The $I_q$ reference value is set to zero to make zero reactive power at the output of the converter. The detailed block diagram is shown in Fig. 2

**Figure 3:**
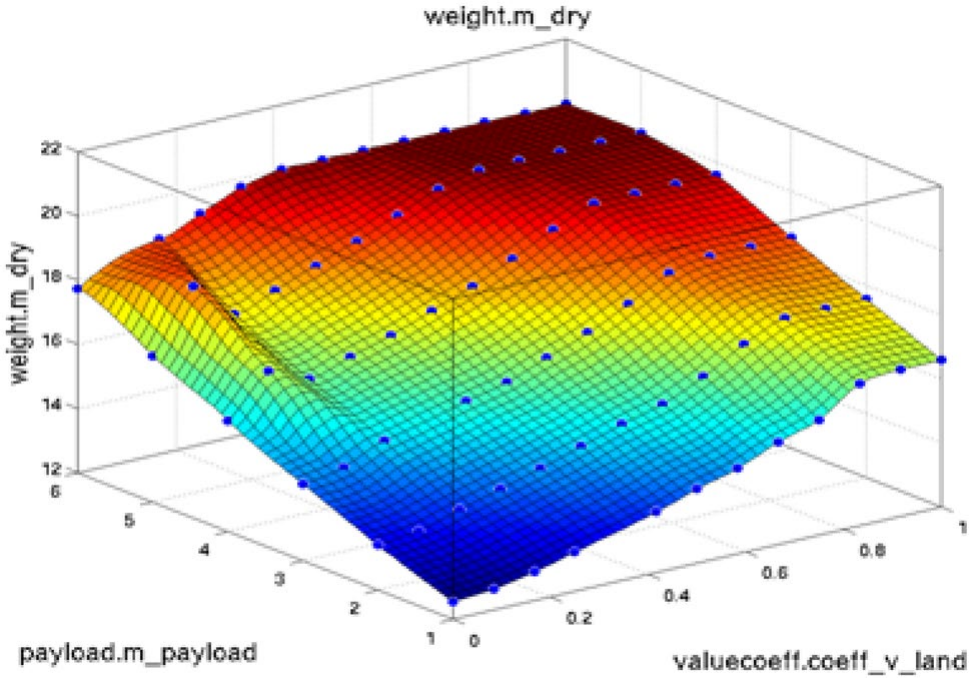
Surface view of coefficient and weight function.

Figure 3 represents the surface view of the co-efficient and weight function used for ANFIS PI controller. As observed the slope surface becomes divergent with increasing wind velocity thereby capturing the maximum data required for calculating the gain parameters. However, if wind velocity increases beyond 20  m/s the slope becomes flattened thereby restricting the maximum wind velocity.

**Figure 4:**
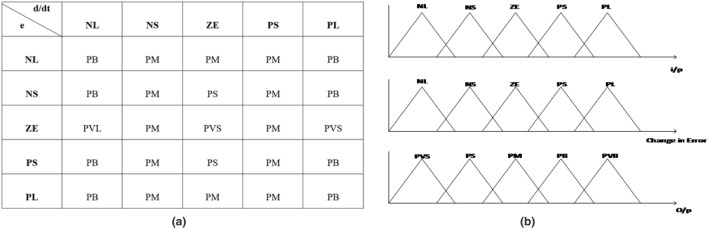
(**a**) Rule table for membership function using IF-THEN statement. (**b**) Triangular membership function for ANFIS PI controller.

**Figure 5:**
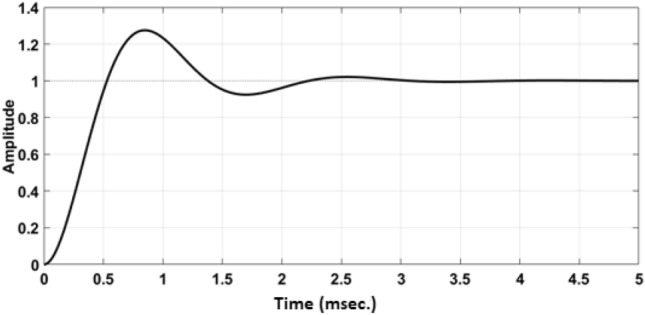
Time response analysis of ANFIS PI controller using step function.

The rule table for the ANFIS PI controller is presented in Fig. 4a. Here altogether 25 situations arise scattering over three different configurations of loading which means the features mostly satisfy the five membership functions in order to be approved for changing the dynamic value of $K_P$ and $K_I$. Figure 5 represents the time response analysis of ANFIS PI controller using a step function. As observed the system has shown only one maximum peak at time 1.2 ms.

**Figure:**
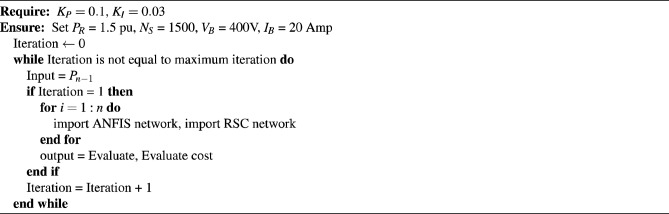
Algorithm 1 Calculate *K*_*P*_ & *K*_I_ perturb ≤ 2 cycles

### ANN controller

The Adaptive Neural Network (ANN) differs from ANFIS in such a manner that the weight function of each hidden layer will be updated automatically based on the training rate of the neural network. The backward sweep along with back propagation provides memory-based intelligence from the training phase starting from output to input.

Here the $d_q$ parameters are used to control the real and reactive components at the converter output and the speed of the DFIG turbine and wind velocity will be taken into consideration for generating the reference voltage. Based on the reference magnitude the coupling capacitor voltage between RSC and GSC will be decided. One of the advantages of this method is, it can track the real-time output at the DFIG converter terminal if and only if during the training and testing condition, the ANN algorithm must satisfy $98\%$ of accuracy and above.

**Figure 6:**
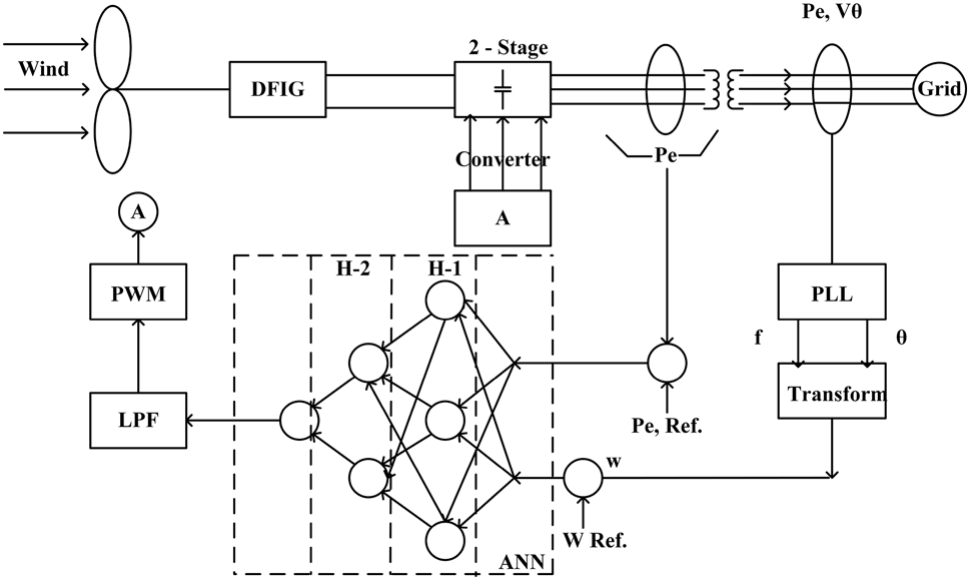
Block diagram of ANN control DFIG grid interconnected system.

The block diagram for ANN ANN-based DFIG controller is presented at Fig. 6. As observed the control algorithm is having three sub-nets, each net corresponds to the reactive power measured at the grid side converter (GSC). Sigmoid-based activation function has been taken into consideration for each hidden layer. The output of the ANFIS PI controller model is given to a low pass filter (LPF) for generating successive PWM firing pulse for the rotor side converter (RSC), which in turn fed to a 2-line stage converter connected in between DFIG and grid.

**Figure 7:**
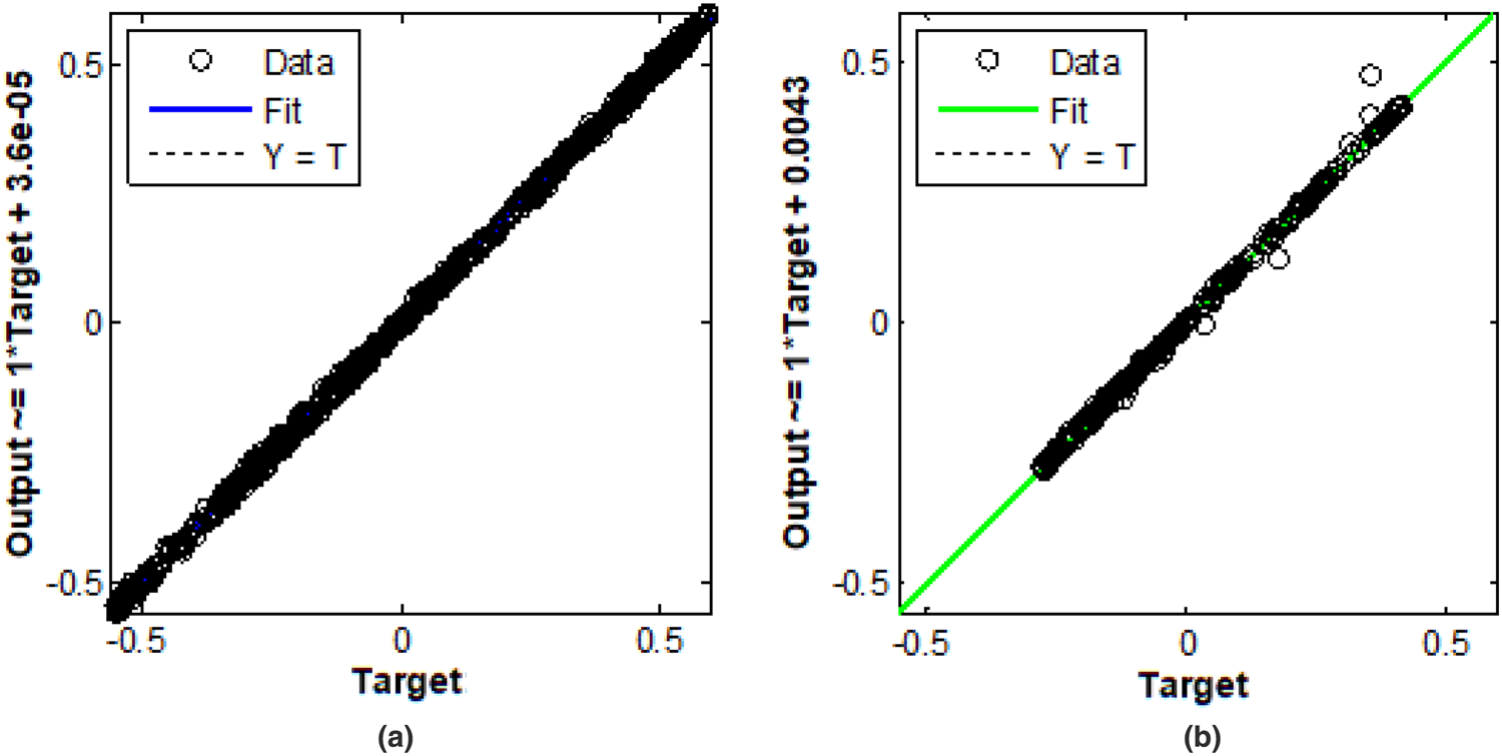
Training and validation of ANN data for $I_d$ and $I_q$ ref. generation, (**a**) Training rate 0.999, (**b**) Training rate 0.998.

**Figure 8:**
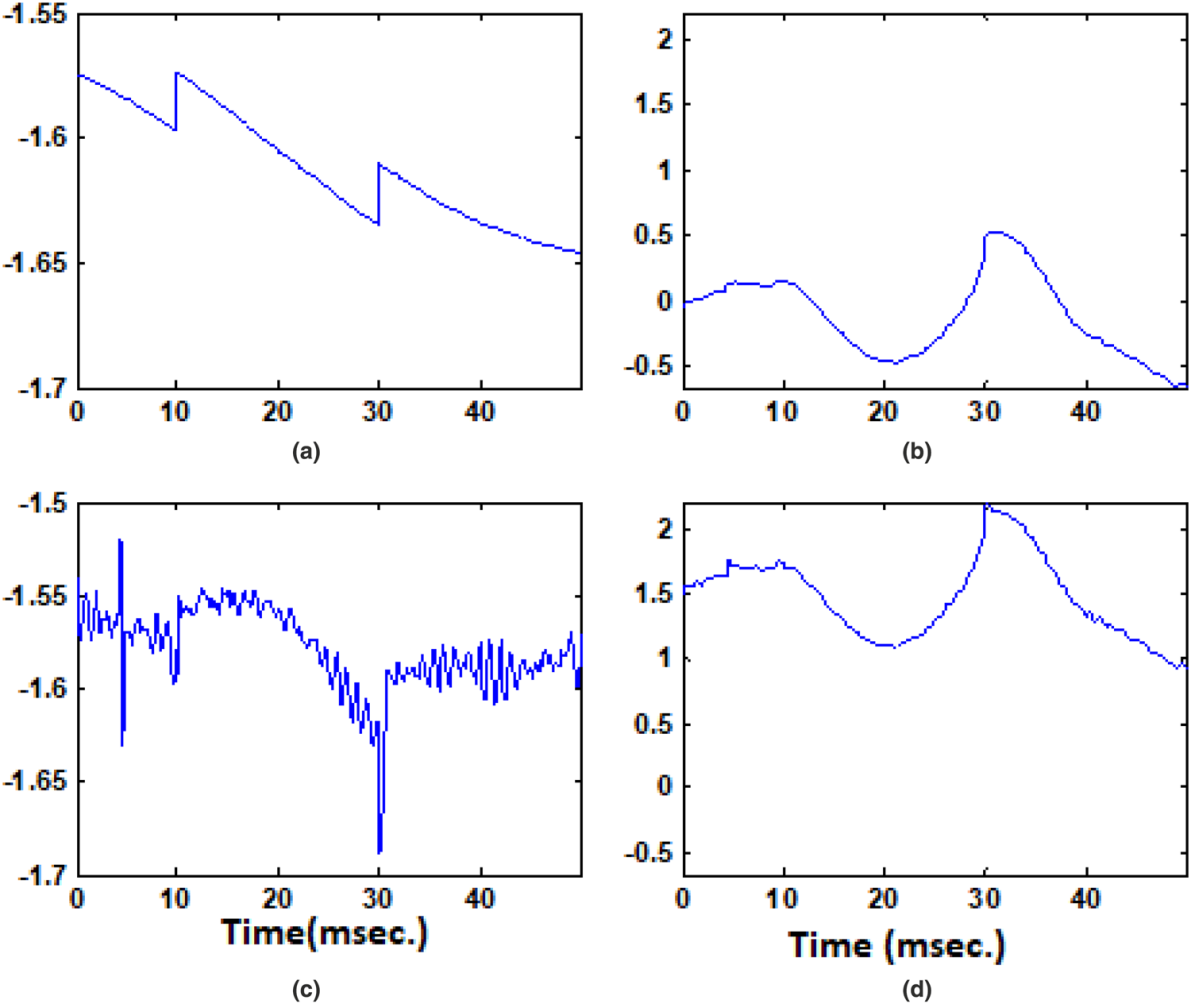
ANN PI controller performance for, (**a**) Input to the controller, (**b**) Output of controller, (**c**) Error rate, (**d**) Neural network output.

Figure 7 represents the training and testing of $I_d$ and $I_q$ data collected as a reference of reactive power from PLL. As observed at Fig. 7a the $I_d$ component has $100\%$ of accuracy in tracking the real power and that of Fig. 7b, the $I_q$ component has $87.33\%$ of accuracy in terms of tracking change in reactive power. The model has been validated for the 70:30 ratio of standard statistical procedure. Figure 8 represents the ANN PI controller performance for (a) Input to controller (b) Output of controller (c) Error rate (d) Neural network output. As observed the error rate up to 30 ms of time is a constant magnitude of -1.55, However after that due to a change in wind gust the error has increased to -1.69 leading to the over-fitting of the curve. At above 30 ms of time, the error rate has decreased to -1.58 and is maintained here after a constant value.

**Figure:**
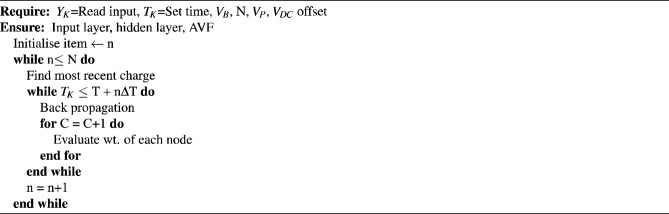
Algorithm 2 Calculate *K*_*P*_ & *K*_I_ perturb ≤ 2 cycles

## Simulation setup

To check the performance characteristic of the proposed algorithm a MATLAB Simulink model has been designed by optimally sizing the internet parameters of various models by the IEEE-1547 grid interconnection protocol. All the interlocking facilities required for the successful operation of DFIG under grid interconnection mode have been provided by the latest state-of-the-art practices for the master trip relay, zone of protection, IP-63 protocol for inverter grid interconnection and IEC codes.

In a DFIG grid interconnected system, the first parameters to be considered are wind turbine parameters along with aerial data. Table 3 represents the wind turbine parameters for MATLAB simulation. Here the air density range has been defined as 1.2–1.25 Kg/m$^3$, thus taking into consideration the effect of temperature on air. However, during the simulation, 1.22 has been taken into consideration. The pitch angle range is given as 0–0.1 Degree, however, during simulation $0^{\circ }$ has been taken into consideration. The radius of the wind blade is 23 mm.

**Table 3:** Wind turbine parameters for MATLAB simulation.

S.N.	Parameters	Rating	Unit
01	Air density	1.2–1.25	$\hbox {kg/m}^3$
02	Pitch angle	0–0.1	°
03	Wind speed	11–17	m/s
04	Radius of swept area	23	NA

After deciding the wind turbine parameters it is required to fix the parameters for DFIG. Therefore Table 4 represents 12 numbers of different DFIG configuration parameters. The stator and rotor power are 60 and 33 KW respectively, with a stator voltage of 400 V or 1 pu has been taken into consideration. Due to 4 number of poles the synchronous speed of the setup is fixed at 1500 RPM with a nominal torque of 238.7  Nm. All types of resistance related to stator and rotor side are fixed below 0.02$\omega$, similarly, all the inductance values are set below 0.01H.

**Table 4:** DFIG parameters considered for simulation in accordance with IEEE 1547.

Sr. No.	Parameters	Rating	Unit
01	Stator pole	4	NA
02	Stator power	60	KW
03	Rotor power	33	KW
04	Stator voltage	400 (1 pu)	V
05	Slip variation	0.2	NA
06	Synchronous speed	1500	RPM
07	Nominal torque	238.7	NM
08	Stator resistance	0.018	$\Omega$
09	Rotor resistance	0.02	$\Omega$
10	Stator inductance	0.01	H
11	Rotor inductance	0.01	H
12	Moment of inertia	18	$\text{kg m}^2$

Again it is required to set the interlocking arrangements for different power quality issues, the RSC and GSC parameters were present in Table 5. As the stator output voltage is 400 V. Therefore the DC reference voltage is kept at 390V and that of capacitor capacitance is set at 500$\mu$F. The grid frequency as per the Indian Standard Code has been fixed by an integrator limiter circuit with a lower boundary of 49.97 to 50.03  Hz. The proportional and integral gain as derived by using the Ziegler–Nichols method is set to 0.82 and 4.2 respectively.

**Table 5:** Simulink model parameters for GSC.

Sr. No.	Parameters	Rating	Unit
01	DC bus rated voltage	390	V
02	DC bus capacitor	500	$\mu$f
03	Grid frequency	49.97–50.03	Hz
04	GSC filter inductance	1.5	mH
05	GSC filter resistance	0.02	$\Omega$
06	Proportional gain	0.82	NA
07	Integral gain	4.2	NA

## Result analysis

In the above section, a detailed simulation setup has been presented by IEEE 1547 standards. Based on the above discussion the optimization model for the proposed algorithm has been designed and presented below.

**Figure 9:**
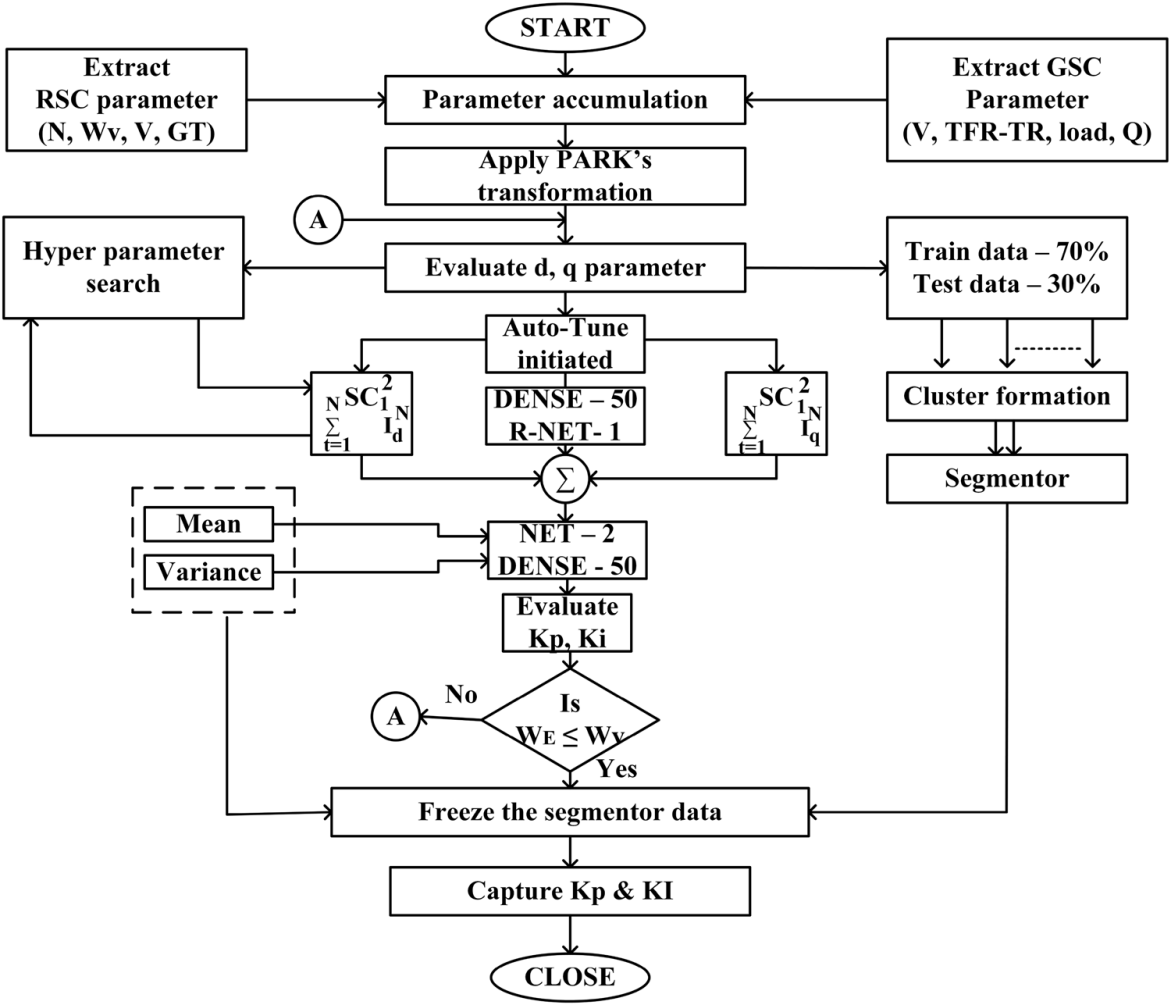
Flowchart for tuning of $K_P$ and $K_I$ by NTM controller (DENSE-50) using RSC and GSC parameters.

Figure 9 represents a flow chart for tuning of $K_P$ and $K_I$ by NTM controller (DENSE-50) using Rotor side controller (RSC) and Grid side controller (GSC) parameters. Four parameters include wind speed (N), wind velocity( $W_v$), voltage at the stator terminal (V) and temperature(GT). Similarly, three parameters from GSC have been recorded, including voltage(V), transformer turns ratio (TFR-TR), and the reactive power demand at the output of GSC. To evaluate the $d_q$ parameters for each data entered into the system a PARK’s Transformation has been applied to the data set to evaluate the $d_q$ parameters separately. To avoid over-fitting each data during the curve fitting analysis, hyper-parameters search space has been set along with the corresponding training and testing data to 70$\%$ and 30$\%$ respectively. Auto-tune has been initiated after the evaluation of $d_q$ parameters through a pre-trained DENSE-50 and the first recurrent neural network set-1 to initiate the tuning procedure. The 2 sets of tuning procedures have been accompanied by 2 different cost minimization functions for evaluating the least error while finding the optimization point. To scale down the error rate both mean and variance have been given to the second R-Net-2 to evaluate and fine-tune the $K_P$ and $K_I$ value. To freeze the $K_P$ and $K_I$ value the cluster segmentation has been carried out by the mean and variance for curve fitting analysis thereby freezing the $K_P$ and $K_I$ for the proposed NTM PI controller. Thus the $K_P$ and $K_I$ become dynamic to the variation either in RSC parameters or GSC parameters respectively. The advantage of the proposed NTM PI controller over all other controllers is that both RSC and GSC parameters have been taken into consideration for deciding the $K_P$ and $K_I$ value using a pre-trained network. However, in other benchmarking models such as PI controller, Fuzzy-PI controller and ANN-PI controller, the effect of both RSC and GSC has not been taken into consideration because of the increase in membership function, hidden layer and time requirement in evaluating the PI controller. The NTM consists of a collection of neural networks, each with a different structure such as recurrent, feedforward, or Convolutional networks. The ensemble architecture of the NTM features dense plexus terminals or dense connections between the layers of the neural network, which facilitate the free flow of information and provide insight into each individual network. During the training phase, the ensemble of neural networks adaptively learns to map input to output by incorporating the ensemble of neural networks into the training data. Each neural network has its own structure, which helps to increase the robustness of the ensemble. Additionally, due to the dense plexus terminals, the ensemble of neural networks improves its performance on unseen data

Table 6 represents the performance analysis of the proposed NTM PI controller during the optimization process at 4 different levels of voltage THD. It is observed that for THD levels of 5.4 and 5.61 with 100 EPOCH levels the mini-batch loss is 0.3. This represents that during the clustering of data, equal segmentation has been applied uniformly throughout the data so that during gradient decent analysis the updation of each weight function has been updated to $30\%$. Similarly at THD level of 5.93, $70\%$ of the cluster weight function is updated. This shows that for a change of $5.7\%$ in the THD level (relative) there is an increased change in THD level of $40\%$. This makes the controller more dynamic to the change in grid parameters. Further, the analysis has been carried out for $6.04\%$ of THD level. However, the mini-batch loss is found to be $20\%$.

**Table 6:** Performance analysis of Proposed NTM-PI controller during Optimization process at four different levels of Voltage THD.

S.N.	THD%	EPOCH	Iteration	Mini batch RMSE	Mini batch loss
01	5.4	1	1	2.09	2.2
		50	50	1.45	1.1
		100	100	0.82	0.3
02	5.61	1	1	2.01	2.0
		50	50	1.29	0.8
		100	100	0.82	0.3
03	5.93	1	1	2.63	3.5
		50	50	1.57	1.2
		100	100	1.16	0.7
04	6.04	1	1	2.38	1.7
		50	50	1.77	0.6
		100	100	1.91	0.2

Table 7 represents performance analysis of the proposed NTM PI controller during the optimization process at 2 different levels of current THD. As observed at a THD level of 8.17 with an EPOCH level of 50 the RMSE is 2.28 and that of 100 EPOCH level is 1.78 respectively. Similarly, at a THD level of 8.23, the RMSE for 50 EPOCH is 2.69 and that of 100 EPOCH is 2.25 respectively. It is also noticed that the mini-batch loss is 1.6 for 100 EPOCH with THD level of 8.17. However, at a THD level of 8.23, a mini-batch loss of 2.5 at 100 EPOCH has been recorded. This means that under current THD the proposed algorithm is overfitted with $16\%$ and $25\%$ respectively, which is $17\%$ less as compared to any other proposed algorithm namely ANN-PI controller.

**Table 7:** Performance analysis of Proposed NTM-PI controller during Optimization process at two different levels of Current THD.

S.N.	THD%	EPOCH	Iteration	Mini batch RMSE	Mini batch loss
01	8.17	1	1	3.11	4.8
		50	50	2.28	2.6
		100	100	1.78	1.6
02	8.23	1	1	3.23	5.2
		50	50	2.69	3.6
		100	100	2.25	2.5

Figure 12 represents a comparative analysis of $V_{DC}$ Ref. voltage waveform for (a) PI controller (b) ANFIS-PI controller (c) ANN-PI controller (d) NTM-PI controller. As observed, in Fig. 12(a) the $V_{DC}$ ref. voltage has undergone a maximum shoot of 1.042 and a maximum variation of 0.97 during the initial transition period. During the second transition event, the minimum voltage is 0.98 pu. By IEEE standard the voltage variation should not increase to below $10\%$ of the reference value or 0.9 pu. In Fig.  12b with ANFIS-PI controller, the maximum overshoot reached 1.041 during the first transient operation whereas during the second transient operation, a minimum value of 0.98 was recorded at 0.15 s. Again, in Fig.  12c, with ANN-PI controller, during the first transient analysis, the maximum overshoot of 1.02 has been noticed with a minimum value of 0.98 and that of Fig. 12d, NTM-PI controller, the maximum overshoot of 1.03 and a minimum overshoot of 0.99 has been observed which is exactly under IEEE and IEC standard. This reveals that the DC ref. voltage requires 0.05 s to settle down to the steady value of 1 pu with two transition oscillations as compared to all other bench-marking models it takes a minimum of four oscillations before getting settled down to 1 pu. Therefore, the proposed algorithm is robust for implementation and practicability.

**Figure 10:**
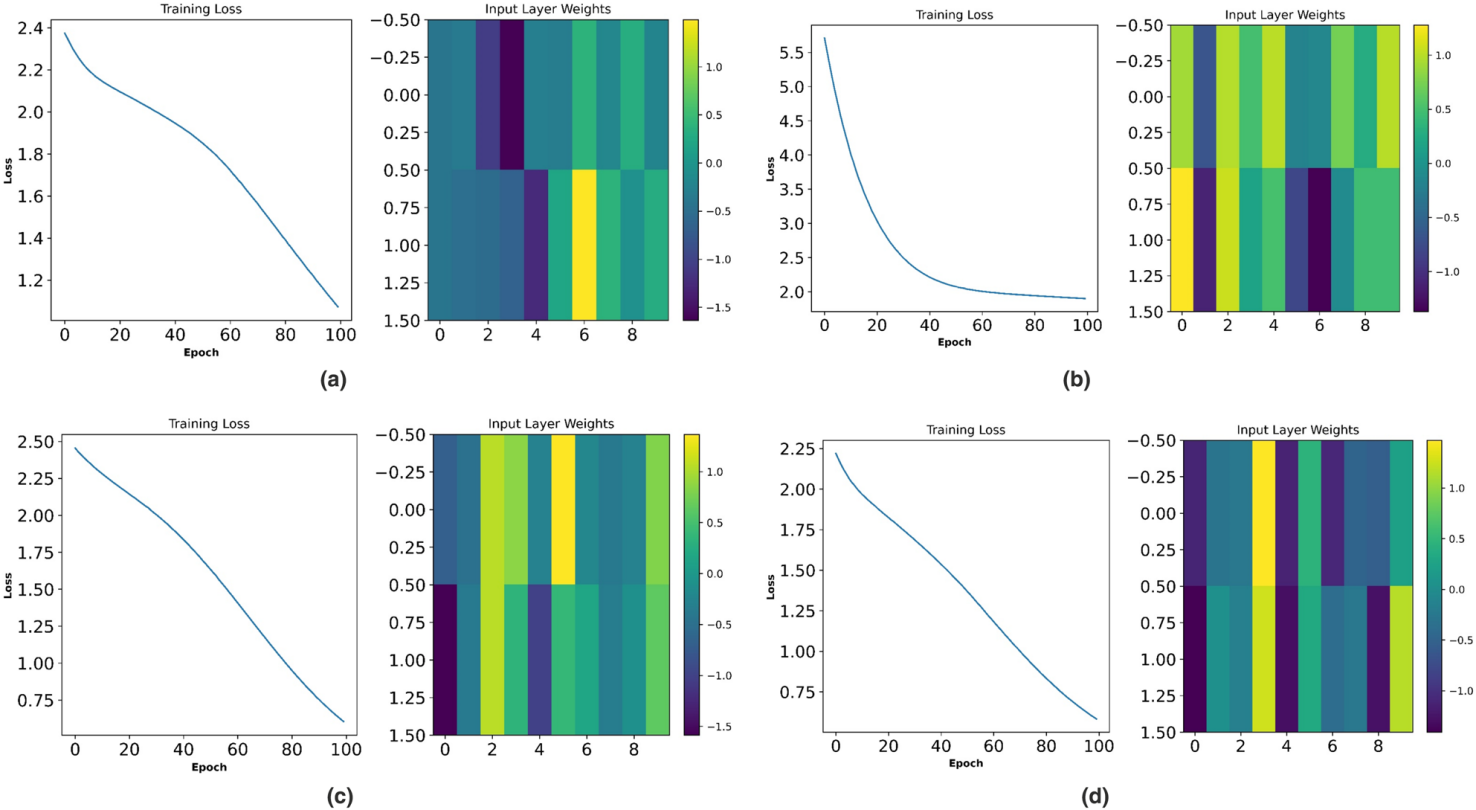
Optimisation process of NTM at, (**a**) ReLu function wt 0.6, (**b**) ReLu function wt 0.73, (**c**) ReLu function wt 0.81, (**d**) ReLu function wt 0.97.

**Figure 11:**
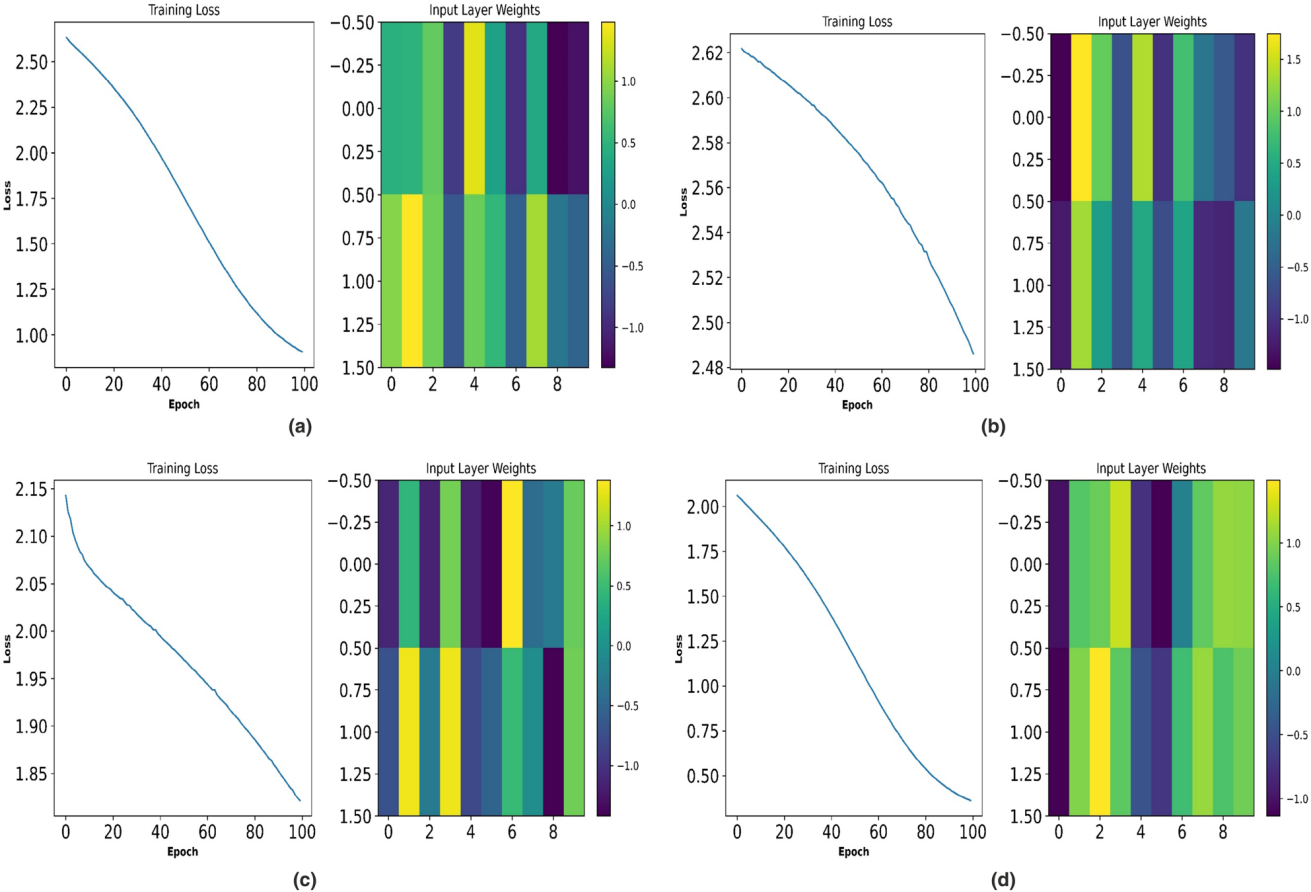
Optimisation process of NTM at, (**a**) Softmax function wt 0.6, (**b**) Softmax function wt 0.73, (**c**) Softmax function wt 0.81, (**d**) Softmax function wt 0.97.

**Figure 12:**
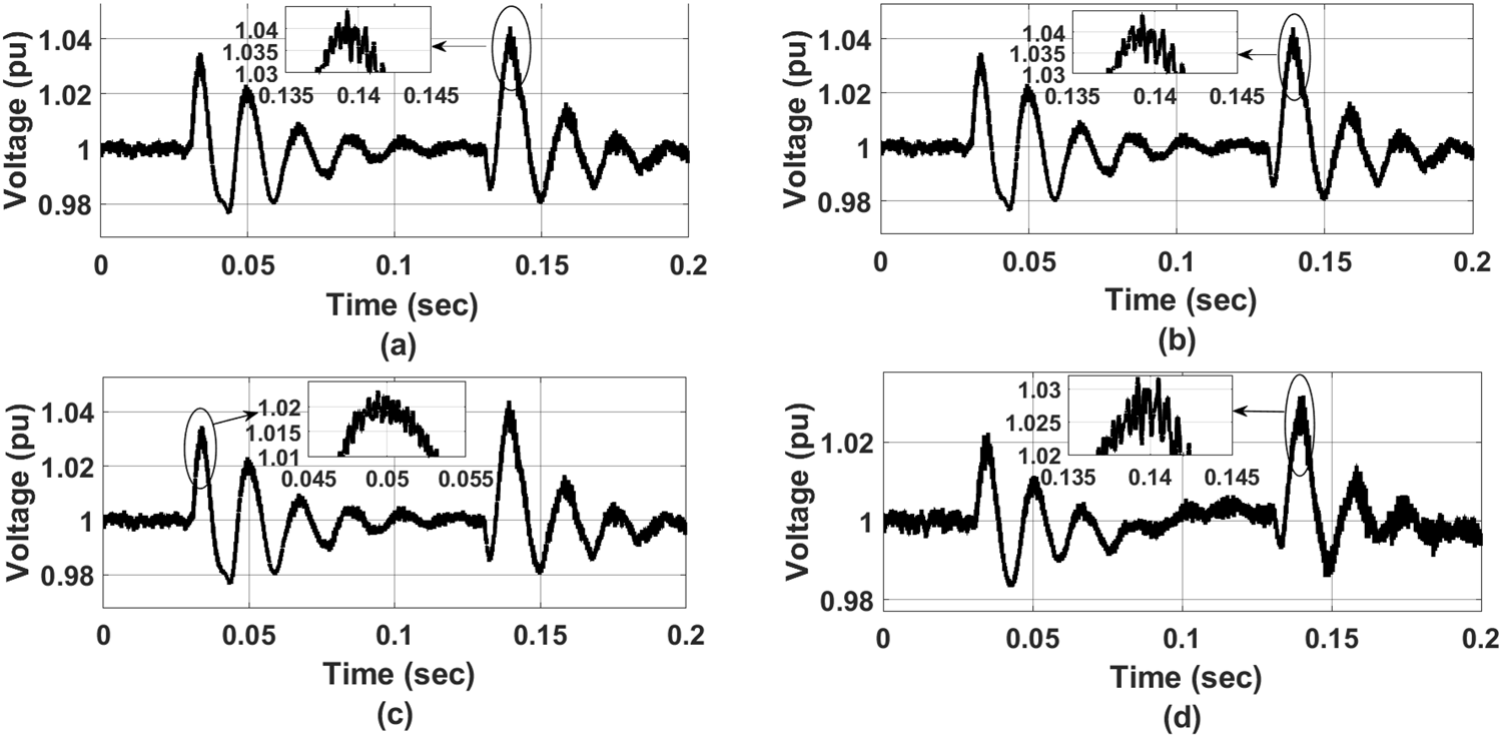
$V_{DC}$ Ref. voltage waveform, (**a**) PI controller, (**b**) ANFIS-PI controller, (**c**) ANN-PI controller, (**d**) NTM-PI controller.

Figures  10 and 11 represent the optimization process of NTM for two different activation functions ReLu and softmax function. As observed, at Fig. 10b the optimization process has undergone an exponential decaying function showing highest possibility of sensitivity to external parameters variation whereas for all other figures of Fig. 10 such as Fig. 10a,c,d, the decaying function is less sensitive to the parameters variation. Therefore, the rest of the research part has been concentrated on ReLu function with activation function wt. value of 0.73. During the softmax optimization process for NTM, it is observed that at Fig. 11c, initially the system has shown an exponential decaying performance up to 18 EPOCH level and thereafter it becomes constantly decaying. In contrast Fig. 11b the system has shown rectangular hyperbola function with a wt value of 0.73 making it suitable for analysis under transient conditions. Again, at Fig. 11a,d, the system has shown a non linear performance throughout the iteration level. Therefore the analysis with NTM has been carried out for Rotor Side Controller (RSC) with ReLu function and for Grid Side Controller (GSC). It is softmax based activation function (Fig. 12).

**Figure 13:**
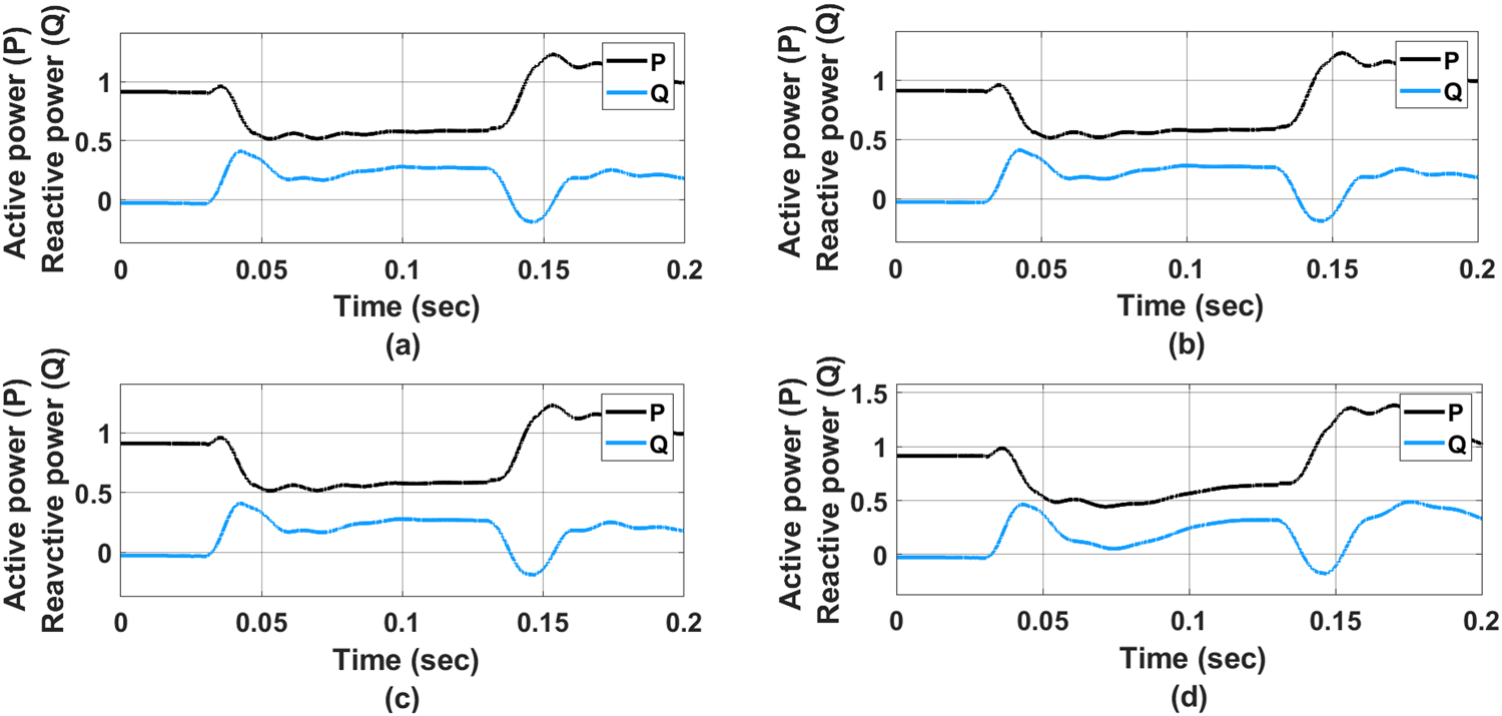
Active and Reactive power flow waveform, (**a**) PI controller, (**b**) ANFIS-PI controller, (**c**) ANN-PI controller, (**d**) NTM-PI controller.

Figure 13 represents the active and reactive power flow waveform for (a) PI controller (b) ANFIS-PI controller (c) ANN-PI controller (d) NTM-PI controller. As observed in Fig.  13a the active power is maintained at 0.95 pu until the first transition occurs at 0.04 s. Here the reactive power has come down to 0.5 pu and it is maintained up to 0.5 pu till 0.14 s. At about 0.15 s the real power has increased to 1.5 pu. Similarly, it is also noticed that the reactive power also shows a similar type of performance before settling to 0.5 pu at the end of 0.15 s. The analysis has also been carried out for Fig. 13b ANFIS-PI controller and that of Fig. 13c ANN-PI controller. In Fig.  13d The NTM-PI controller analysis has been presented. However, the real power is maintained approximately at 1pu before undergoing a transition. Due to the use of a real-time dynamic PI controller, the performance of the active power is quite better as compared to other benchmarking models. During this time the reactive power has also increased to 0.5 pu from 0 pu before reaching o.45  pu at 0.2  s. The proposed NTM-PI controller is more robust as compared to all other benchmarking controllers in terms of tracking the real-time variation in the DFIG output.

**Figure 14:**
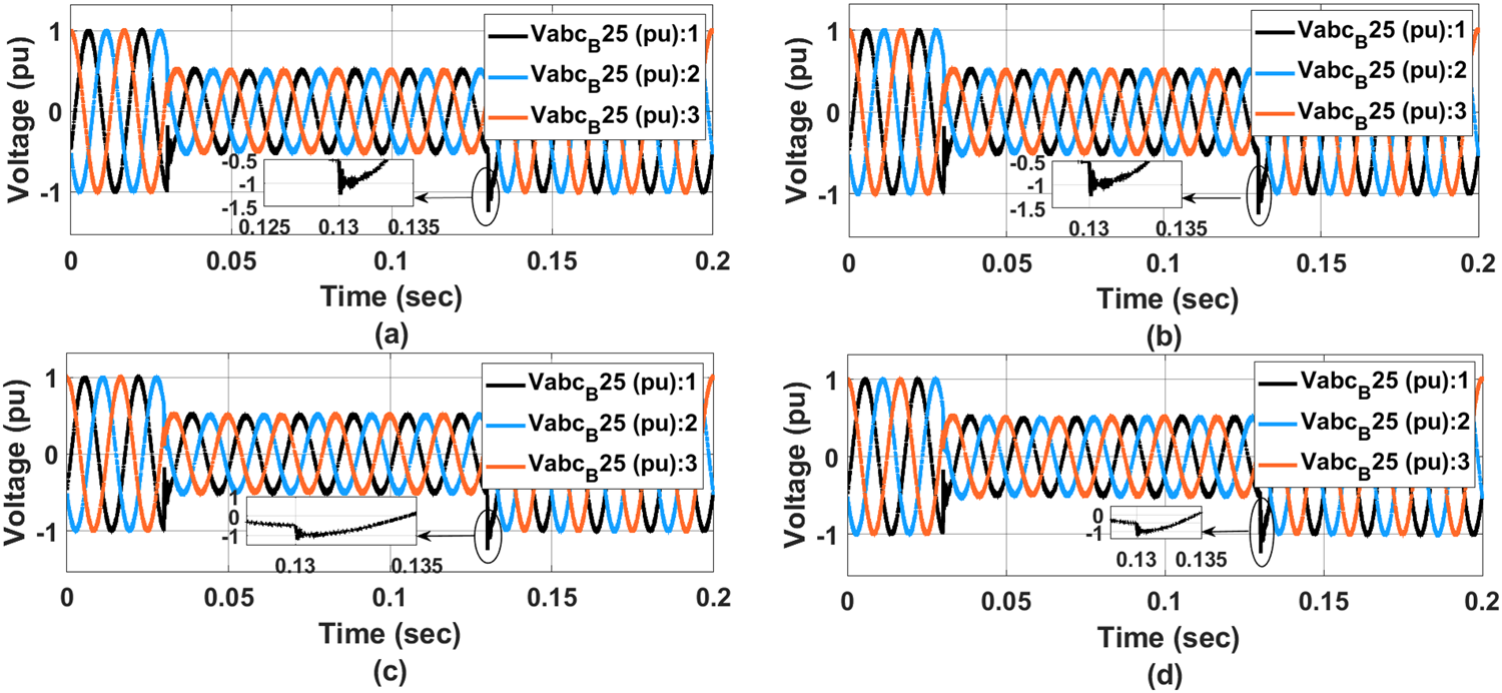
$V_{abc}$ voltage at $B_{25}$ waveform, (**a**) PI controller, (**b**) ANFIS-PI controller, (**c**) ANN-PI controller, (**d**) NTM-PI controller.

Figure 14 represents the $V_{abc}$ voltage at the transformer for (a) PI controller (b) ANFIS-PI controller (c) ANN-PI controller (d) NTM-PI controller. As noticed, during the transition condition, the output voltage waveform with PI controller has shown two different transitions during loading and on-loading transformer at 0.04 and 0.014 s respectively. At 0.14 s, the PI controller-based waveform (Fig. 14a) showed three different sub-synchronous oscillations for two cycles before settling down to a THD level of 5. Similarly in Fig. 14b the ANFIS-PI controller is presented however for the same type of fault condition, the voltage has undergone two cycles of oscillations before reaching the final steady state. In Fig. 14c, the voltage waveform is almost oscillating by the loading pattern. However, the system has undergone a maximum undershoot of $-$ 1.1 pu in the second transition effect. As compared to the ANFIS-PI controller here the oscillation is limited to 2.5 cycles. With the proposed NTM-PI controller model, a maximum undershoot of $-$ 1.1 pu has been achieved with a maximum deviation of 0.5 cycles. From the above voltage waveform analysis, it is noticed that the effect of different loading patterns can be analysed with the half cycle of the voltage waveform with the proposed NTM-PI controller.

**Figure 15:**
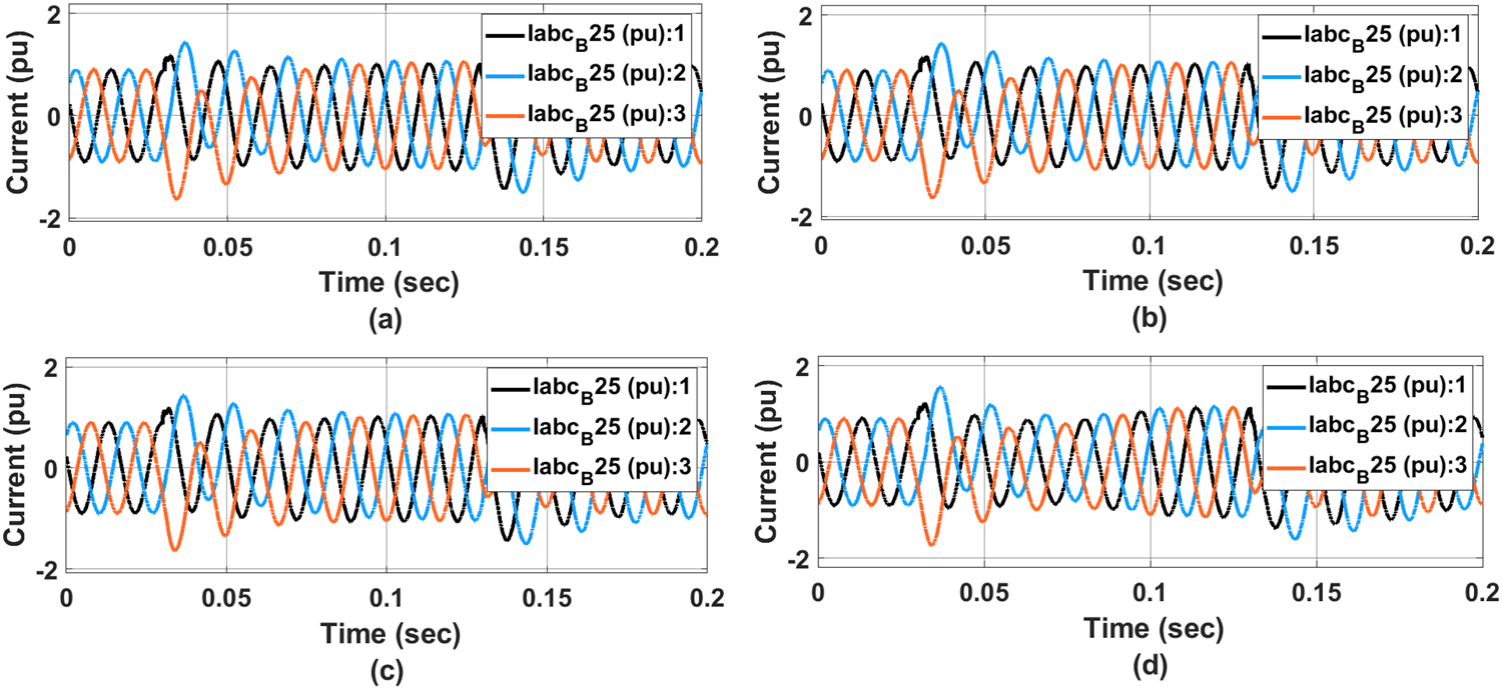
$I_{abc}$ current at source side waveform, (**a**) PI controller, (**b**) ANFIS-PI controller, (**c**) ANN-PI controller, (**d**) NTM-PI controller.

Figure 15 represents $I_{abc}$ current waveform at source side bus (a) PI controller (b) ANFIS-PI controller (c) ANN-PI controller (d) NTM-PI controller. As observed with the PI controller during the initial stage of load transition, the grid has undergone a voltage swell of $13.6\%$. The synchronization between all three phases occurs after three cycles of disturbance. Similarly, with ANFIS-PI controller the disturbance was noticed for three cycles with a maximum deviation of voltage swell is $13.88\%$. Here the system is brought back into its initial condition after three cycles of disturbances. With a proposed NTM-PI controller the voltage swell goes up to $11.03\%$ and synchronisation between all three phases occurs within two cycles of distribution.

**Figure 16:**
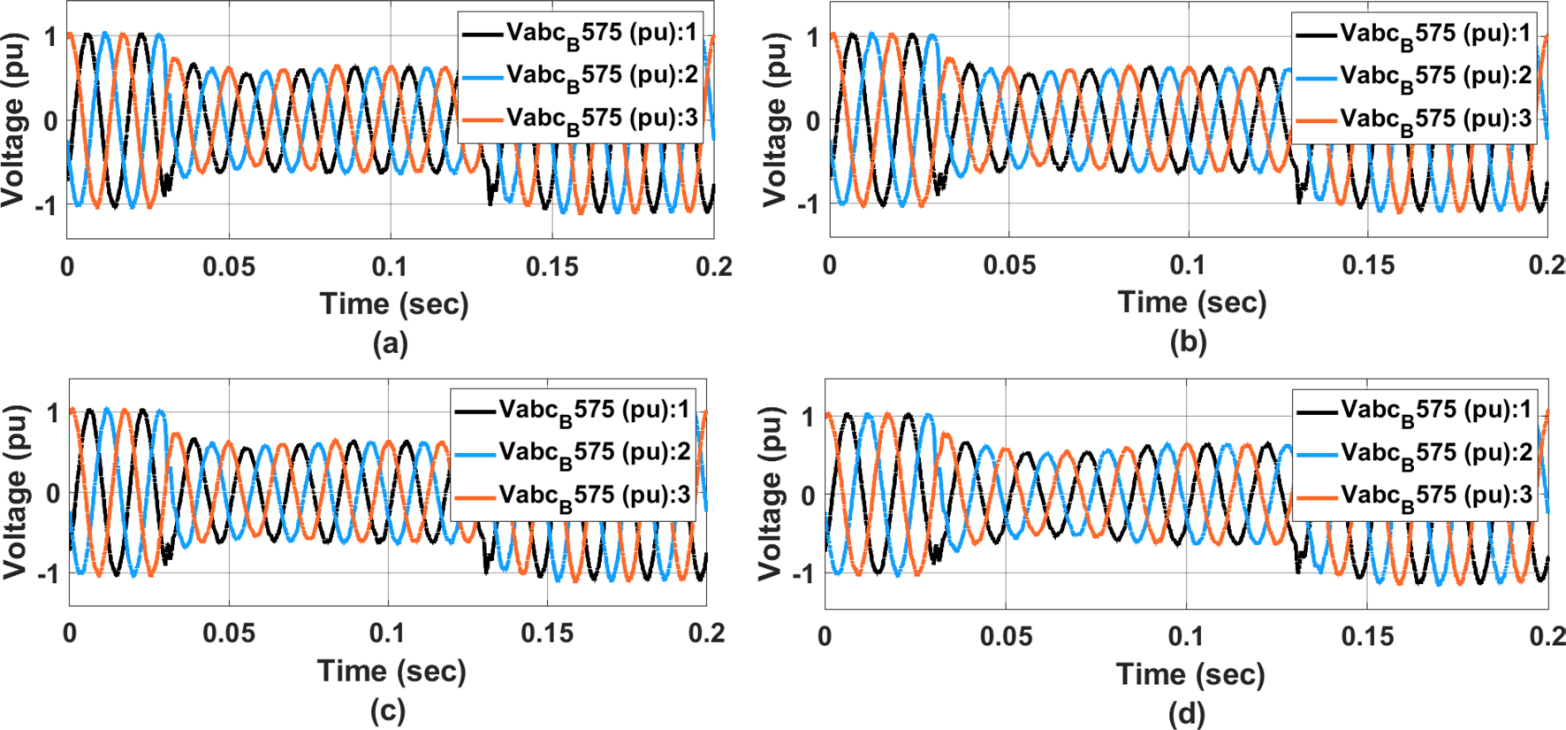
$V_{abc}$ voltage at $B_{575}$ waveform, (**a**) PI controller, (**b**) ANFIS-PI controller, (**c**) ANN-PI controller, (**d**) NTM-PI controller.

Figure 16 represents $V_{abc}$ voltage at $B_{575}$ waveform (a) PI controller (b) ANFIS-PI controller (c) ANN-PI controller (d) NTM-PI controller. As observed with PI controller voltage near the PCC has undergone a voltage sag of $4\%$ for three cycles. However, in Fig. 16b with ANFIS-PI controller the voltage disturbance has undergone up to $4.2\%$ (voltage sag). This effect has been noticed upto 0.1 s thereby making it unsuitable for real-time implementation. To overcome that problem, the system has been modeled with the proposed NTM-PI controller and hence the voltage disturbance settled down within 0.04 s from the instant of the occurrence of the fault. It is also noticed that the wave-top has undergone a single transition event before settling into its normal state. This may be due to the variation in the load at the rate of $2.67\%$. All the IEEE and IEC standards have been validated concerning the voltage and current waveform as presented in Figs. 15 and 16 respectively.

The $R^2$ error determines the fitting of the model at different parameter variations for the specified algorithm. A higher value of $R^2$ error represents the model is best fitted to the data and it has taken $90\%$ of data during regression analysis. The $R^2$ error is evaluated along with variance.

Table 8 represents the performance comparison analysis of different algorithms during $\pm 10\%$ of voltage fluctuation at PCC. 7 parameters such as $R^2$, RMSE, MAE, Accuracy, Sensitivity, PPV and NPV of different bench-marking controllers such as PI-controller, ANFIS-PI, ANN-PI and NTM-PI are compared for $\pm 10\%$ of voltage fluctuation at point of common coupling. As observed for $10\%$ of voltage fluctuation the $R^2$ value of PI controller is 0.641 and for ANFIS-PI is 0.776 respectively. Similarly, for ANN-PI and NTM-PI, it is 0.863 and 0.911. RMSE of the PI controller will be 0.098 and for ANFIS-PI it will be 0.081, whereas for ANN-PI and NTM-PI the values will be 0.083 and 0.067 respectively. MAE of the PI controller is 1.178, whereas for ANFIS-PI it is 1.124. Similarly, for ANN-PI and NTM-PI it is 1.993 and 0.816. During $\pm 10\%$ of voltage fluctuation at PCC the accuracy and sensitivity of the controller varies. The accuracy of the PI controller will be $81.177\%$ and for ANFIS-PI it is $85.091\%$. $91.279\%$ and $93.268\%$ will the accuracy of ANN-PI and NTM-PI. Similarly, the sensitivity of the controller is compared, where for the PI controller the sensitivity is $83.234\%$, ANFIS-PI sensitivity $\%$ is 84.003, ANN-PI sensitivity will be $86.418\%$ and NTM-PI sensitivity is $89.546\%$. Here the PPV in $\%$ is also compared given the values are $91.017\%$ for the PI controller, $93.271\%$ for ANFIS-PI. $92.079\%$ of ANN-PI and $93.587\%$ for NTM-PI. Furthermore NPV in $\%$ is also a parameter which is compared for different controllers. As observed the NPV$\%$of PI controller is 92.109, whereas for ANFIS-PI it is $91.312\%$. Similarly, NPV$\%$ values for ANN-PI and NTM-PI are $93.45425\%$ and $92.173\%$ respectively.

**Table 8:** Performance comparison analysis of different algorithm during $\pm 10 \%$ of voltage fluctuation at PCC.

Parameter	PI—controller	ANFIS-PI	ANN-PI	NTM-PI
$R^2$	0.641	0.776	0.863	0.911
RMSE	0.098	0.081	0.083	0.067
MAE	1.178	1.124	1.993	0.816
Accuracy ($\%$)	81.177	85.091	91.279	93.268
Sensitivity ($\%$)	83.234	84.003	86.418	89.546
PPV ($\%$)	91.017	93.271	92.079	93.587
NPV ($\%$)	92.109	91.312	93.425	92.173

Table 9 represents the performance comparison analysis of different algorithms during $\pm 5\%$ of voltage fluctuation at PCC. 7 parameters such as $R^2$, RMSE, MAE, Accuracy, Sensitivity, PPV and NPV of different benchmarking controllers such as PI-controller, ANFIS-PI, ANN-PI and NTM-PI are compared for $\pm 5\%$ of voltage fluctuation at point of common coupling. As observed for $5\%$ of voltage fluctuation the $R^2$ value of PI controller is 0.800 and for ANFIS-PI is 0.813 respectively. Similarly, for ANN-PI and NTM-PI it is 0.891 and 0.930. RMSE of PI controller will be 0.132 and for ANFIS-PI it will be 0.147, whereas for ANN-PI and NTM-PI the values will be 0.097 and 0.073 respectively. MAE of PI controller is 1.461, whereas for ANFIS-PI it is 1.169, similarly, for ANN-PI and NTM-PI it is 2.018 and 0.833. During $\pm 5\%$ of voltage fluctuation at PCC the accuracy and sensitivity of the controller also varies. The accuracy of PI controller will be $79.564\%$ and for ANFIS-PI it is $81.163\%$, similarly $92.197\%$ and $94.206\%$ will be the accuracy of ANN-PI and NTM-PI. The sensitivity of the controller is compared, where for the PI controller the sensitivity is $87.41\%$, ANFIS-PI sensitivity $\%$ is 87.021, ANN-PI sensitivity will be $87.287\%$ and NTM-PI sensitivity is $90.447\%$. Here the PPV in $\%$ is also compared given the values are $91.93\%$ for PI controller, $94.938\%$ for ANFIS-PI. $94.013\%$ of ANN-PI and $94.996\%$ for NTM-PI. Furthermore NPV in $\%$ is also a parameter which is compared for different controllers. As observed the NPV of PI controller is $93.96\%$, whereas for ANFIS-PI it is $92.687\%$. Similarly, NPV$\%$ values for ANN-PI and NTM-PI are $94.832\%$ and $93.101\%$ respectively.

**Table 9:** Performance comparison analysis of different algorithm during $\pm 5 \%$ of frequency regulation at PCC.

Parameter	PI-controller	ANFIS-PI	ANN-PI	NTM-PI
R$^{2}$	0.800	0.813	0.891	0.930
RMSE	0.132	0.147	0.097	0.073
MAE	1.461	1.169	2.018	0.833
Accuracy($\%$)	79.564	81.163	92.197	94.206
Sensitivity($\%$)	87.41	87.021	87.287	90.447
PPV($\%$)	91.93	94.938	94.013	94.996
NPV($\%$)	93.96	92.687	94.832	93.101

**Figure 17:**
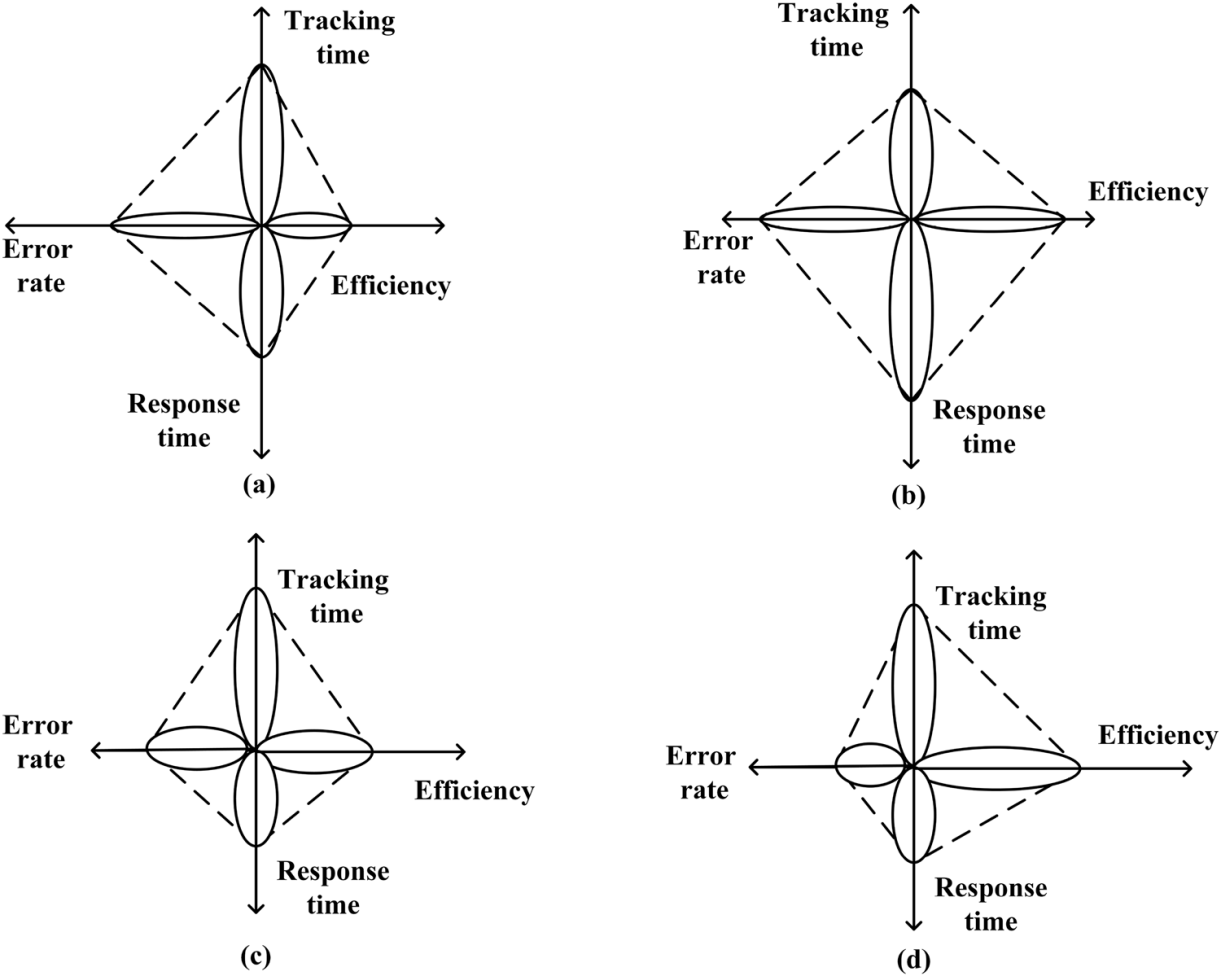
Performance comparison Tracking time, error rate, efficiency and response time, (**a**) PI controller, (**b**) ANFIS—PI controller, (**c**) ANN—PI controller, (**d**) NTM—PI controller.

Figure 17 represents Performance comparison Tracking time, error rate, efficiency and response time (a) PI controller (b) ANFIS-PI controller (c) ANN-PI controller (d) NTM-PI controller. As observed in Fig. 17d, the efficiency has increased for the NTM-PI controller and at the same time it takes lesser response time and also low error rate as compared to all other bench-marking controllers. This clearly states that the proposed NTM-PI controller is good in terms of tracking time, efficiency and response rate to any loading condition at the point of common coupling.

One potential application of this research in real-world power grids is its utility in parameter settling under dynamic load-changing patterns. It has been observed that the NTM-PI controller exhibits a maximum overshoot of only 8.23% and a settling time of 0.93 s, respectively. Furthermore, it aids in the analysis and continuous updating of control policies with the assistance of real-time data. Traditional methods commonly used for controlling systems in real-world power grids often struggle with non-linear dynamics. The NTM-PI controller possesses the capability to adjust and learn the non-linearities associated with the control method. Additionally, this method demonstrates quick adaptability within the control system, where the system is continuously updated with real-time data

## Conclusion

In this research article, an improved grid interconnection of DFIG has been presented by using the conditional Neural Tuning Method as an escort to the classical PI controller for improving the controllability of the controller.

During the analysis, it is observed that most of the PI controllers face problems in parameter settling under dynamic load-changing patterns. Here the dynamic load changing refers to the loading effect of the grid on the DFIG. In order to avoid such a scenario in this paper a novel practice has been adapted by making the $K_P$ and $K_I$ as dynamic quantities. During the controllability analysis of the proposed NTM-PI controller and the bench-marking controller, it is observed that NTM-PI controller has less maximum overshoot and less settling time of $8.23\%$ and 0.93sec respectively. It has also been noticed that with the ANFIS and ANN controllers, it is very difficult to achieve the statistical performance of the above-stated controller with all other benchmarking models.

At Tables 6 and 7, the performance analysis of the proposed NTM-PI controller has been presented with different voltage and current THD. It is found that the mini-batch loss at 100 EPOCH value is marginal and controllable by effectively updating the $90\%$ of the system weight function in the hidden layers. All these analyses have been carried out in a controlled environment and strictly restricted to unconstrained optimization. The implementation of DPT enhances the learning capabilities of the neural network which further leads to development of accurate learning capabilities in the PI controller. The present study has clearly demonstrated the stability of the model and power quality using grid interconnection.

As an extension to the present research adaptive learning techniques can be adapted with an Intelligent Electronic Device (IED) to make the system more robust which can bring down the disturbance up to half a cycle at the point of observation. Cyber-physical-based layer for gateway application may be enabled for bidirectional communication between the operator and the controller. Further, the research can also be taken up by considering some other system parameters such as wind speed variations, power system imbalances and different grid code compliances such as IEEE, IEC and IS-Code. By interconnecting these elements into the training phase of neural network would enable the further development of adaptive PI controller which will further enhance the stability of the system.

## Data Availability

The data used to support the findings of this study are included in the article.
